# Developing a real-time detection tool and an early warning score using a continuous wearable multi-parameter monitor

**DOI:** 10.3389/fphys.2023.1138647

**Published:** 2023-03-29

**Authors:** Arik Eisenkraft, Nir Goldstein, Roei Merin, Meir Fons, Arik Ben Ishay, Dean Nachman, Yftach Gepner

**Affiliations:** ^1^ Biobeat Technologies Ltd., Petach Tikva, Israel; ^2^ Faculty of Medicine, Institute for Research in Military Medicine, The Hebrew University of Jerusalem, Israel Defense Force Medical Corps, Jerusalem, Israel; ^3^ Heart Institute, Hadassah Medical Center, The Hebrew University of Jerusalem, Jerusalem, Israel; ^4^ Department of Epidemiology and Preventive Medicine, School of Public Health, Sackler Faculty of Medicine and Sylvan Adams Sports Institute, Tel Aviv University, Tel Aviv, Israel

**Keywords:** early warning score (EWS), patient deterioration, prevention, alarm fatigue, pre-symptomatic detection, multi-parameter monitoring

## Abstract

**Background:** Currently-used tools for early recognition of clinical deterioration have high sensitivity, but with low specificity and are based on infrequent measurements. We aimed to develop a pre-symptomatic and real-time detection and warning tool for potential patients’ deterioration based on multi-parameter real-time warning score (MPRT-WS).

**Methods:** A total of more than 2 million measurements were collected, pooled, and analyzed from 521 participants, of which 361 were patients in general wards defined at high-risk for deterioration and 160 were healthy participants allocation as controls. The risk score stratification was based on cutoffs of multiple physiological parameters predefined by a panel of specialists, and included heart rate, blood oxygen saturation (SpO_2_), respiratory rate, cuffless systolic and diastolic blood pressure (SBP and DBP), body temperature, stroke volume (SV), cardiac output, and systemic vascular resistance (SVR), recorded every 5 min for a period of up to 72 h. The data was used to define the various risk levels of a real-time detection and warning tool, comparing it with the clinically-used National Early Warning Score (NEWS).

**Results:** When comparing risk levels among patients using both tools, 92.6%, 6.1%, and 1.3% of the readings were defined as “Low”, “Medium”, and “High” risk with NEWS, and 92.9%, 6.4%, and 0.7%, respectively, with MPRT-WS (*p* = 0.863 between tools). Among the 39 patients that deteriorated, 30 patients received ‘High’ or ‘Urgent’ using the MPRT-WS (42.7 ± 49.1 h before they deteriorated), and only 6 received ‘High’ score using the NEWS. The main abnormal vitals for the MPRT-WS were SpO_2_, SBP, and SV for the “Urgent” risk level, DBP, SVR, and SBP for the “High” risk level, and DBP, SpO_2_, and SVR for the “Medium” risk level.

**Conclusion:** As the new detection and warning tool is based on highly-frequent monitoring capabilities, it provides medical teams with timely alerts of pre-symptomatic and real-time deterioration.

## Introduction

Increasing life expectancy with its associated co-morbidities grow the number, complexity, and acuity of patients (Case and Deaton, 2017). Healthcare providers need objective methods and medical tools to identify and quantify clinical deterioration. This could help preventing crisis and hospital admission or providing early alerts within the hospital setting (Phillips, 2021). Several hospital practices are directed towards intervening before clinical deterioration events occur, as patients usually develop physiological instability preceding such events (Hillman et al., 2002; Kause et al., 2004; Fu et al., 2020). As a result, early warning scores (EWS) have been developed to assist healthcare providers (HCP) in recognizing initial signs of deterioration, allowing timely and prioritized intervention (Wuytak et al., 2017; Phillips, 2021). Usually, EWS takes routinely-measured physiological measurements as input and evaluates the patient’s risk of clinical deterioration as output, defined in a score. A set threshold level is also provided, and if and when a patient’s score passes this certain threshold, an alert is sent to the HCP for further evaluation and intervention (Fu et al., 2020). EWS used in hospitals can predict the risk of early and in-hospital mortality (at 24, 48, and more than 72 h), as well as hospital and intensive care unit (ICU) admission (Arévalo-Buitrago et al., 2021). One of the most recognized tools for the identification of deterioration in acute settings is the National Early Warning Score (NEWS). Some known setbacks of the NEWS and other scales are the frequency of scoring and response, integration into practice, a miscalculation by healthcare providers, and perceived dissonance with clinical judgment. Benefits include communication and prioritization of care as well as sensitivity (though this varies between studies), particularly in predicting poor outcomes (Wuytak et al., 2017; Phillips, 2021). Moreover, there is a need for better tools that will provide early pre-symptomatic detection of deterioration in various patient populations.

We have previously shown that a remote patient monitoring (RPM) platform can provide early pre-symptomatic detection of flu (Goldstein et al., 2021) and detect the risk of patient deterioration many hours before it happens in the general ward (Itelman et al., 2022).

In this study, we aimed to develop a pre-symptomatic and real-time detection and warning tool with risk scores of potential patients’ deterioration, based on multi-parameter real-time warning score (termed “MPRT-WS”).

## Materials and methods

### Study design and overview

Deidentified physiological and clinical data were collected and pooled from two groups of participants. The first group included 410 patients that were recruited for a study aimed to look at the early detection of deterioration among patients at high risk within general wards (MOH_2020-07-12_009133; NCT04220359) (Itelman et al., 2022). The second group included 160 healthy individuals that were recruited for a study looking at side effects of the Pfizer COVID-19 vaccine (TAU_0002522-1) (Gepner et al., 2022).

Within the vaccine study on healthy individuals, data were collected automatically for five consecutive days, 1-day pre- and 4 days post-vaccination. We included in the current analysis only the 24 h before vaccination, as it was defined as a baseline period with no external intervention. There were no cases of clinical deterioration among this group. The data collected from this group served as a normal basis for the analysis and score. Among the high-risk patients, 49 subjects were hospitalized for less than 24 h, leading to their exclusion. A total of 361 patients were included. Clinical deterioration among this group was defined based on the ABCDE criteria (Smith and Bowden, 2017) and included also the need for cardiopulmonary resuscitation, transfer to an intensive care unit, or death, during their hospitalization (more details included in Itelman et al., 2022). In this group of patients, the physiological data from the wearable monitors have been collected automatically during the first 72 h from admission. The monitoring platform did not collect any personally identifiable information (PII), and only the serial numbers of the devices were used to pair a device to a person, a task performed by the HCPs only.

### The remote patient monitoring device

Frequent intermittent monitoring was achieved using a wireless, wearable, non-invasive, reflective PPG-based chest patch monitor (BB-613WP, Biobeat Technologies Ltd., Petah Tikva, Israel) (Nachman et al., 2020a; Atzmon et al., 2020; Nachman et al., 2020b; Bar-On et al., 2021; Eisenkraft et al., 2021). The data was automatically transmitted to a cloud-based web platform repository. Each monitor collected a set of physiological parameters every 5 min, and this included heart rate (HR), blood oxygen saturation (SpO_2_), respiratory rate (RR), cuffless blood pressure (BP) including systolic BP (SBP), and diastolic BP (DBP), body temperature, stroke volume (SV), cardiac output (CO), and systemic vascular resistance (SVR). The chest patch monitor incorporates several sensors to provide the various measurements. A proprietary PPG sensor measures heart rate and SpO2 in a similar way other pulse oximeters provide these parameters. On top of that, by using pulse wave transit time and pulse wave analysis, it also measures cuffless blood pressure, respiratory rate, and stroke volume. Based on these measurements, the company’s algorithms calculate the cardiac output and the systemic vascular resistance. A non-invasive thermistor embedded in the chest patch measures body temperature based on the heat flux approach (Double Sensor) (Janke et al., 2021).

### The currently used National Early Warning Score

**FIGURE 1:**
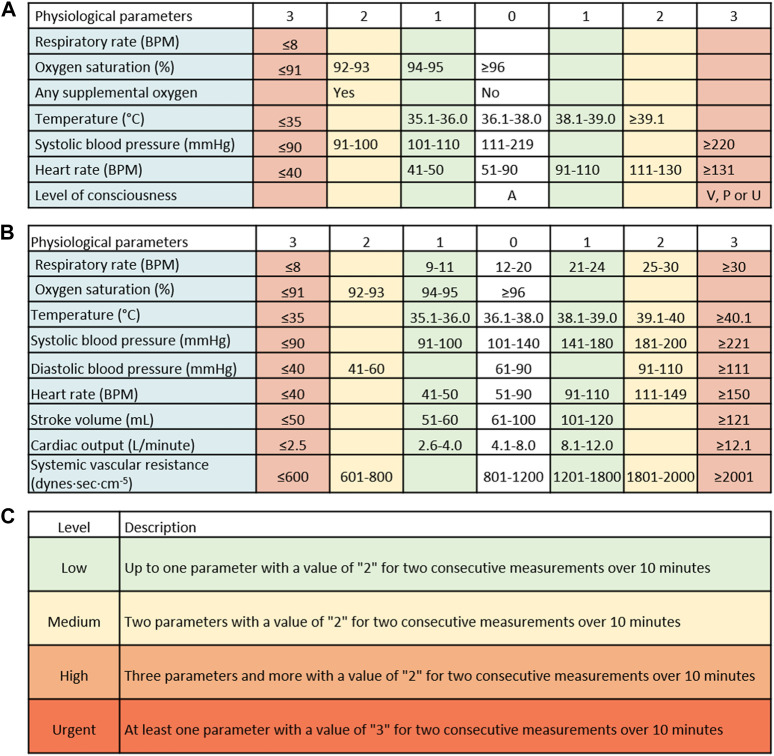
The currently-used National Early Warning Score (NEWS) and the new tool. **(A)** The NEWS score is comprised of systolic blood pressure, heart rate, oxygen saturation, respiratory rate, and temperature, each with a defined risk level ranging from 0 to 3. Another feature is defined as the “Red score”, which should result in an immediate check-up of the patient, though the score is not provided in real-time. **(B)** The new score includes 9 vital signs: systolic blood pressure, diastolic blood pressure, heart rate, oxygen saturation, respiratory rate, temperature, stroke volume, cardiac output, and systemic vascular resistance, each with a defined risk level ranging from 0 to 3. **(C)** The new early warning score integrates the risk levels of each parameter with a time element, i.e., at least two consecutive measurements over a 10-min period. RPM–respirations per minute; BPM–beats per minute.

The NEWS scale was previously described and is among the most commonly-used EWS systems in the world (Alam et al., 2015; Bilben et al., 2016; Ehara et al., 2019; Credland et al., 2021). The NEWS scale was used in this study as a control system, to compare its output with the output of the tested tool. Whenever the physiological parameters were collected, a NEWS value was automatically generated and transferred by the same monitoring system. Importantly, in a real-world setting, the vitals comprising the score—SBP, HR, RR, SpO_2_, and temperature—are collected infrequently, usually by nurses, which then manually calculate the score, mark whether the patients receive supplemental oxygen and their level of consciousness, and enter the resulting value into the electronic medical record (EMR). The NEWS scale includes three levels of alert—“Low”, “Medium”, and “High” (Figure 1A). Each level entails a different intervention by the HCPs. Notably, the vitals that are included in the NEWS calculation are usually taken once a shift, while during this study, measurements were taken intermittently at a higher frequency (Itelman et al., 2022).

### Defining a new pre-symptomatic and real-time detection and warning tool (“MPRT-WS”)

Based on the frequent prospective measurements collected by the wearable monitoring devices, a new risk score was retrospectively formulated. The aim was to allow a pre-symptomatic and real-time detection and warning tool, providing additional capabilities to other existing EWS tools. In a previous study, it was shown that frequent measurements of NEWS did not improve its sensitivity and specificity (Itelman et al., 2022). The newly-developed MPRT-WS was based on nine physiological parameters, including the five parameters on which the NEWS scale is based (SBP, HR, RR, SpO_2_, and temperature), as well as DBP, SV, CO, and SVR. As the monitoring platform provides intermittent readings and frequent scoring, we have also added the dimension of time into the scoring approach. The process was conducted by an expert panel in the field of cardiology, internal medicine, human physiology, and acute/intensive care. We started by using accepted normal ranges for each parameter (Diepenbrock, 2015; Jones and Fix, 2015; Burns and Delgado, 2019; Urden et al., 2020), together with values gathered from all of the participants, including healthy and sick individuals, to define the normal limits and the various limits of concern for each of the nine parameters. Each parameter was scored individually (see an example in Supplementary Figure S1). We also used the NEWS approach of defining four score levels (0–3) for each parameter. As a general rule, the accepted normal ranges for each parameter received a score level of “0”, followed by a score level of “1”for a slight change from the normal range and up to a score level of “3”for a change that might result in immediate risk. We did not include the need to add input on oxygen supplements and the level of consciousness following the Alert, Voice, Pain, Unresponsive (AVPU) scale, as used in the NEWS system. Next, we aggregated the score of each parameter, leading to the level of alert. Moreover, the system was developed to provide two components: a detection component, aiming to provide an immediate alert of an imminent deterioration in patients’ clinical condition, and a second component providing a score, warning of the potential to deteriorate, similar to an early warning score–yet provided in a continual and automated manner.

### Statistical analysis and data presentation

Results are presented as mean ± standard deviation. Independent samples *t*-test was used for between-groups comparison, and Levene’s test was used for equality of variance. Chi-square test was used to compare the distribution of the risk values. Fisher’s exact test was used to compare the number of clinical deterioration events identified by the two scores. Sensitivity was calculated as the number of patients with clinical deterioration identified by the system, divided by the number of patients with clinical deterioration identified by the clinical stuff. All tests were 2-tailed, and significance was defined as *p* < 0.05. Statistical analysis was performed using IBM SPSS ver. 25. Analysis and visualization of each parameter were performed using PowerBI and Python 3.8 with the following libraries: Pandas, Numpy, Scipy, Seaborn, and Matplotlib.

## Results

A total of more than 2 million measurements were collected and analyzed in this work. The demographic characteristics of the participants are shown in Table 1. Among the 361 patients included in the final analysis, 39 experienced clinical deterioration, with the currently-used NEWS system providing a high alert score (7 and above or at least one parameter with a value of ‘Red score’) in 6 of them. None of the patients received oxygen supplements, nor there was any documentation of deterioration based on the AVPU scale, during the monitoring period. By analyzing the data from all of the participants, including both the controls and the patients, and by looking at the normal ranges for each parameter, we have reached a scoring scale for each included parameter (Figure 1B). Next, we integrated the score of each parameter with the number of consecutive repeats of the same score in a given period and defined the new warning score tool (Figure 1C). The frequent collection of vitals allowed us to add a dimension of urgency to the new tool. Thus, and unlike in the NEWS scale, the new MPRT-WS tool include four levels of alerts—“Low” level, with up to one parameter with a value of “2”; “Medium” level, with two parameters with a value of “2”; “High” level, with three parameters and more with a value of “2”; and an “Urgent” level, including at least one parameter with a value of “3”. For each level, the time element included in the definitions was based on at least two consecutive measurements with abnormal levels over 10 min, assuring those abnormal readings were not the result of a coincidental event. “Urgent” is not part of the EWS score, as it warrants an immediate response by the healthcare providers.

**TABLE 1:** Demographic data of the participants. BMI, body mass index; SPO2, blood oxygen saturation. ns, not significant.

	Healthy		Hospitalized						
	(*n* = 160)		(*n* = 361)						
Age (years)	72 ± 15.3		69.0 ± 15.5						
Sex (m/f)	75/85		211/150						
BMI (kg/m2)	24.9 ± 4.4		27.0 ± 5.3			Healthy vs. Hospitalized			

	Day	Night		Day	Night		Day		Night
SpO2 (%)	96.9 ± 0.8	96.5 ± 1.1		94.3 ± 5.9	93.8 ± 5.8		<0.0001		<0.0001
Respiratory rate (breath/min)	15.9 ± 0.9	15.8 ± 1.5		17.1 ± 2.1	17.0 ± 2.1		<0.0001		<0.0001
Temperature (C°)	37.5 ± 0.4	37.3 ± 0.4		37.4 ± 0.5	37.3 ± 0.5		0.015		0.207
Heart rate (bpm)	80.9 ± 9.0	69.8 ± 10.0		77.7 ± 14.8	74.7 ± 15.0		0.003		<0.0001
Systolic blood pressure (mmHg)	128.2 ± 14.7	121.3 ± 14.3		133.4 ± 24.0	132.0 ± 23.9		0.003		<0.0001
Diastolic blood pressure (mmHg)	82.1 ± 9.4	76.6 ± 9.7		71.0 ± 13.7	69.7 ± 13.5		<0.0001		<0.0001
Pulse pressure (mmHg)	46.2 ± 9.9	44.7 ± 9.6		62.4 ± 20.8	62.3 ± 20.7		<0.0001		<0.0001
Mean arterial pressure (mmHg)	97.5 ± 10.4	91.5 ± 10.5		91.8 ± 14.9	90.5 ± 14.7		<0.0001		ns
Stroke volume (mL)	80.7 ± 10.3	77.0 ± 10.2		73.9 ± 14.3	73.0 ± 14.0		<0.0001		<0.0001
Cardiac output (L/min)	6.6 ± 0.9	5.4 ± 1.0		5.7 ± 1.0	5.4 ± 1.1		<0.0001		ns
Cardiac index (L/min/m2)	3.6 ± 0.7	3.0 ± 0.7		3.1 ± 0.7	2.9 ± 0.7		<0.0001		ns
Systemic vascular resistance (dynes/sec/cm−5)	1,240.8 ± 184.0	1,422.1 ± 257.4		1,352.3 ± 293.3	1,405.4 ± 314.5		<0.0001		ns

**FIGURE 2:**
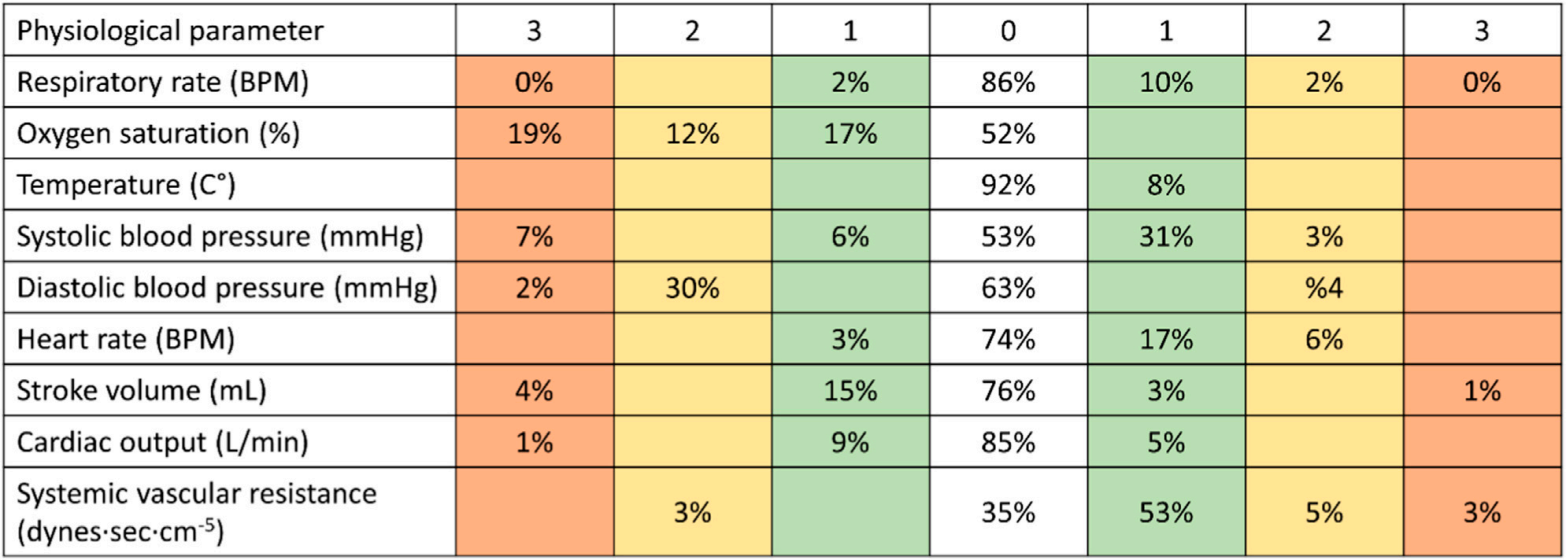
Analyzing data of patients using the new tool. After the definitions of the new score were in place, we tested to see if they helped with the detection of patient deterioration. When using the new tool, most of the readings were in the range of “0”and “1”scores (80%–100% in the various physiological parameters), with only a low rate of “2”and “3”scores. RPM–respirations per minute; BPM–beats per minute.

After the definitions were in place, we tested to see if they helped with the detection of patient deterioration (Figure 2). We found that in the patient population included in the study, most of the readings were in the range of “0”and “1”scores, with only a low rate of “2”and “3”scores (Figure 2).

**FIGURE 3:**
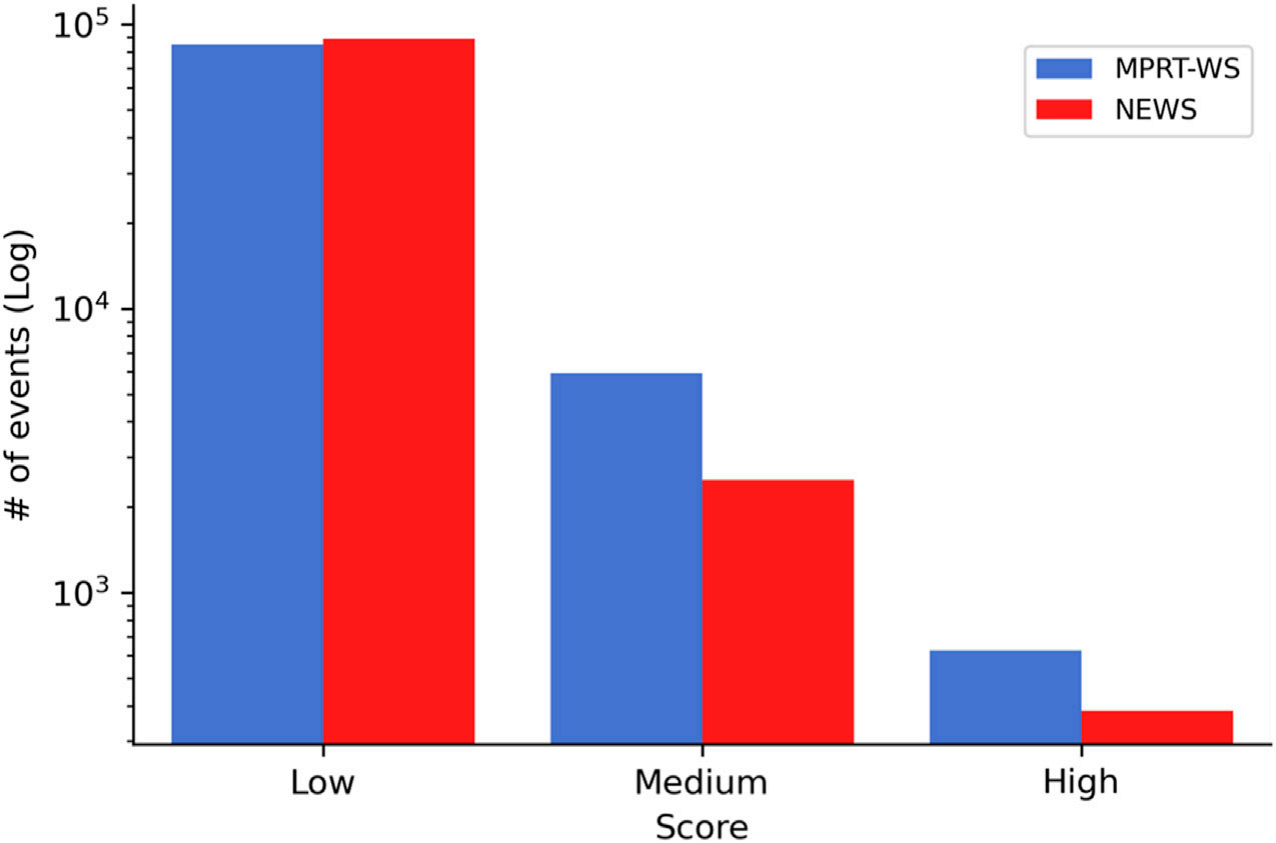
Comparing the risk score readings between the new alert tool and the currently-used NEWS system. Included are readings from the patients (*n* = 361).

When comparing the MPRT-WS with the currently-used NEWS system using the data of the patients (*n* = 361), we found “Low” risk values in 92.9% of readings vs. 92.6% in the NEWS, “Medium” risk was 6.4% of the readings vs. 6.1% in the NEWS, and 0.7% were defined at “High” risk, vs. 1.3% in the NEWS. When looking at the number of events, we found 85,247“Low” events with the MPRT-WS vs. 108,782 with NEWS, 5,924“Medium” events with MPRT-WS vs. 7,158 with NEWS, and 624“High” events with MPRT-WS vs. 1,491 with NEWS (Figure 3). The distribution of the risk values as defined by the MPRT-WS was not different from the distribution of risk values defined by NEWS (*p* = 0.863). We also looked at adding the temporal component to the currently-used NEWS system, to see if this alone has an influence on the NEWS score, and found that there were 111,931 (95.3%) “Low” risk values, 4,755 (4.1%) “Medium” risk events, and 744 (0.6%) “High” risk events.

On top of these risk readings, the system also detected 25,635 readings (21.8% of the total readings) defined as “Urgent”, readings that would provide real-time warning of a deterioration that is happening at that moment. Since the NEWS does not provide a real-time alert of such deterioration, we did not have any values to compare this with.

**FIGURE 4:**
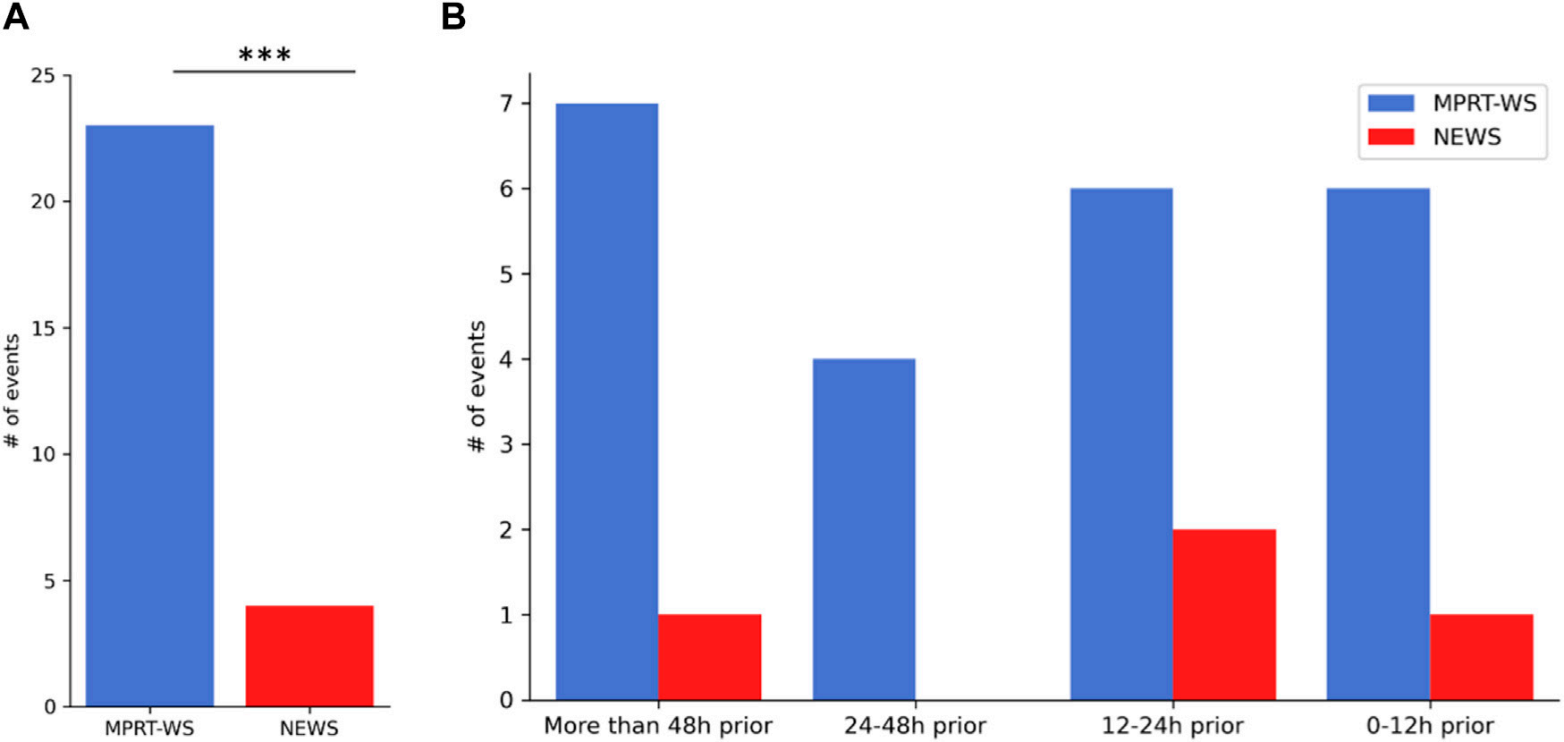
The number of “High” and “Urgent” events detected using the new warning tool before overt clinical deterioration. 39 patients had clinical deterioration. 30 patients of them received ‘High’ or ‘Urgent’ using the MPRT-WS, and in 23 **(A)** patients of these 30 that initiated an alarm, the system identified it 42.7 ± 49.1 h before they deteriorated **(B)**. 6 of the patients with clinical deterioration received ‘High’ score using the NEWS, and in 4 of them **(A)**, the system identified it 40.5 ± 52.9 h before they deteriorated, when frequently collected using the monitoring platform **(B)**.

Among the patients that deteriorated (*n* = 39), 30 patients received ‘High’ or ‘Urgent’ using the MPRT-WS, and in 23 (Figure 4A) patients of these 30 that initiated an alarm, the system identified it 42.7 ± 49.1 h before they deteriorated (Figure 4B). Among the patients with clinical deterioration, 6 received ‘High’ score using the NEWS, and in 4 of them (Figure 4A), the system identified it 40.5 ± 52.9 h before they deteriorated, when frequently collected using the monitoring platform (Figure 4B). The sensitivity was 59% for MPRT-WS, 10% for NEWS, and 5% for NEWS with the temporal component.

**FIGURE 5:**
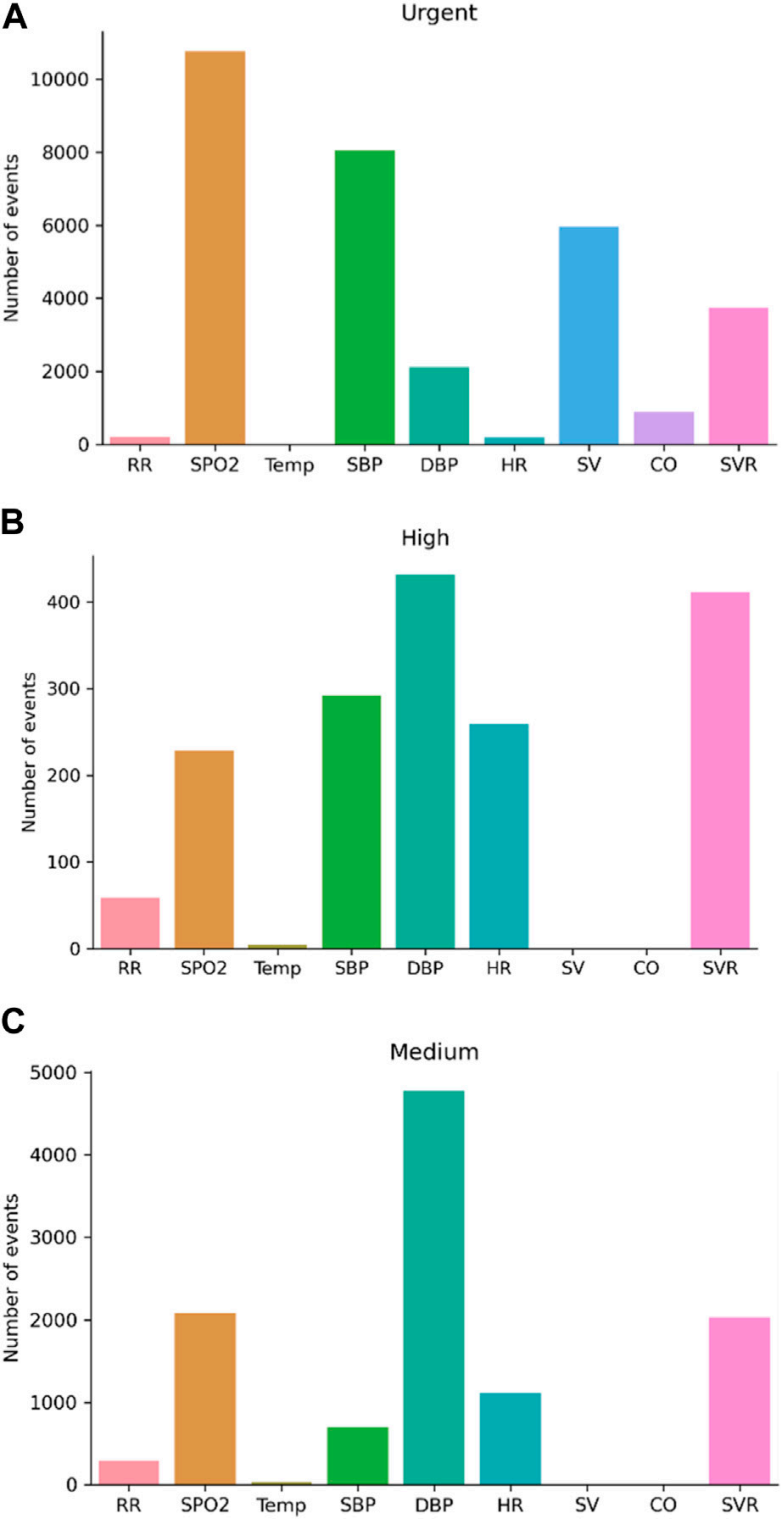
Changes from normal values in the different score levels. **(A)** Urgent score; **(B)** High score; **(C)** Medium score. CO, cardiac output, SV, stroke volume, SpO_2_ blood oxygen saturation, and systolic blood pressure (SBP) increase, while the weight of diastolic blood pressure (DBP) and systemic vascular resistance (SVR) decreases when compared with “high” and “medium” risk levels; RR, respiratory rate; HR, heart rate; Temp, temperature. **(A)**.

Next, we looked at which of the parameters showed changes from normal values in the different levels (Figure 5). This allowed us to see which of the vital signs were the most abnormal at each level. Interestingly, in the “Urgent” level, the main abnormal vitals were SpO_2_, SBP, and SV (Figure 5A). In the “High” level these included DBP, SVR, and SBP, and in the “Medium” risk level it was DBP, SpO_2_, and SVR (Figures 5B, C, respectively). The temperature was not a leading vital in any of the levels and almost absent from the “Urgent” level.

**FIGURE 6:**
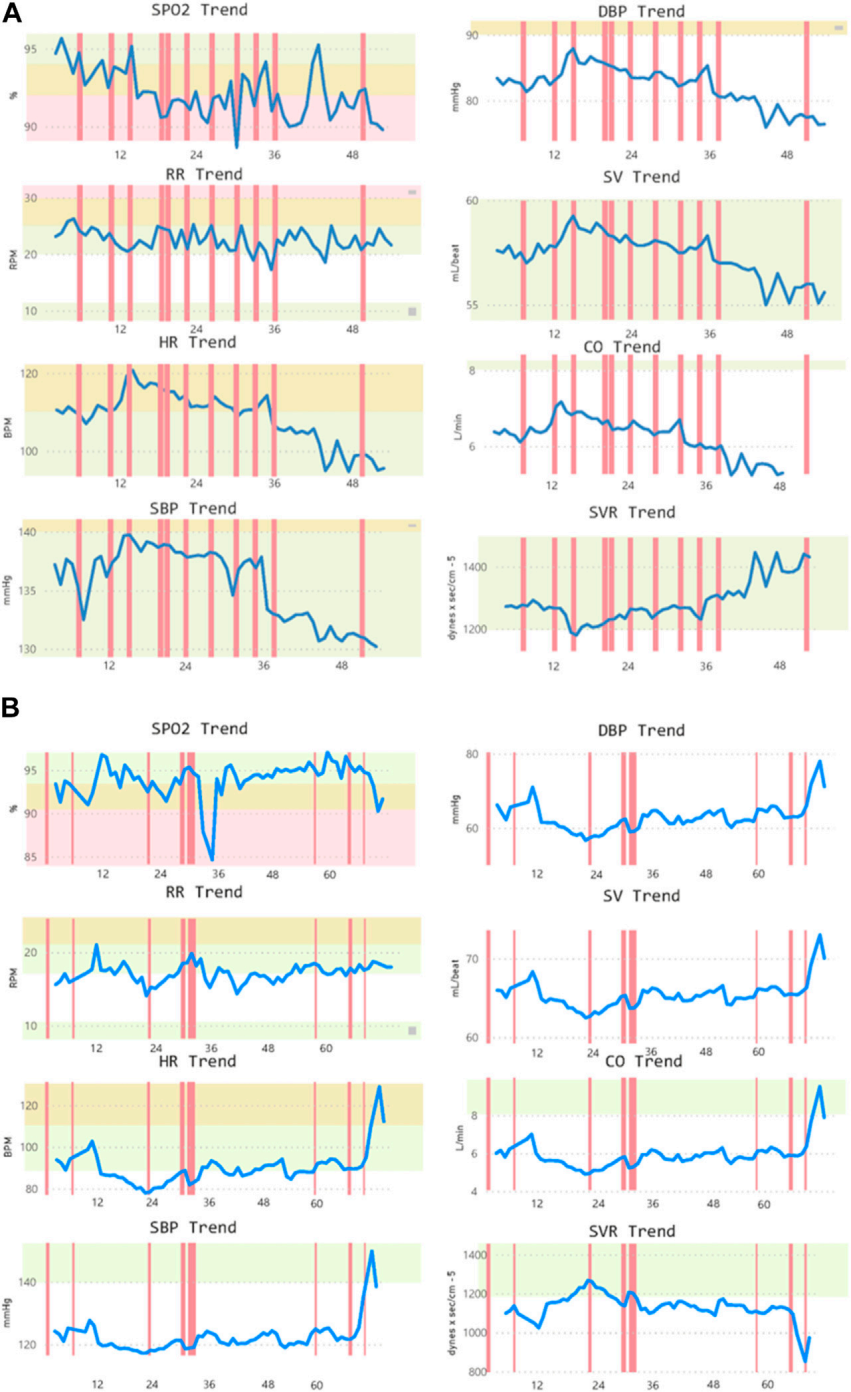
Two samples of admitted patients with **(A)** terminal cancer and **(B)** sepsis. In both samples, alerts are shown based on the new detection tool. CO, cardiac output; DBP, diastolic blood pressure; HR, heart rate; RR, respiratory rate; SBP, systolic blood pressure; SpO_2_, blood oxygen saturation; SV, stroke volume; SVR, systemic vascular resistance.

Figure 6 provides an example of two admitted patients, one with terminal cancer and one with sepsis, showing the scores provided when using the MPRT-WS tool. In the septic patient, the currently-used NEWS scale produced 66 events defined as “Low”, 169 events defined as “Medium”, and 444 events defined as “High”. The MPRT-WS tool produced 205 events defined as “Low”, 124 events defined as “Medium”, 187 events defined as “High”, and 163 events defined as “Urgent”. In the terminal cancer patient, the currently-used NEWS scale produced 622 events defined as “Low”, 120 events defined as “Medium”, and 7 events defined as “High”. The MPRT-WS tool produced 636 events defined as “Low”, 49 events defined as “Medium”, 20 events defined as “High”, and 44 events defined as “Urgent”.

**FIGURE 7:**
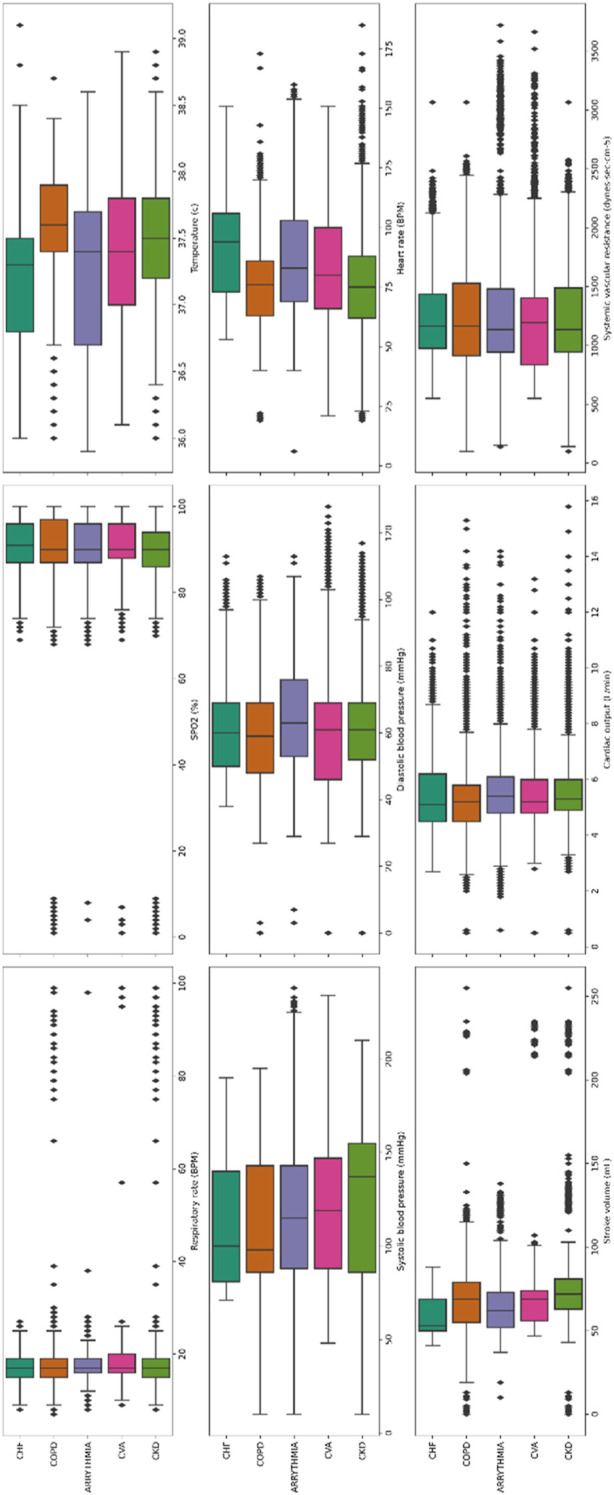
Segmentation of “High” or “Urgent” alerts in each physiological parameter in groups of patients based on the diagnosis on admission. 3,881 readings in patients with congestive heart failure (CHF, *n* = 24); 3,976 readings in patients with cerebrovascular accident (CVA, *n* = 29); 4,844 readings in patients with chronic obstructive pulmonary disease (COPD, *n* = 39); 5,258 readings in patients with chronic kidney disease (CKD, *n* = 53); 9,727 patients with arrhythmias (*n* = 67). The boxes in the current figure, represent the inter-quartile range (IQR, between percentile 25 and 75). The line in the middle is the median value. The whiskers are 1.5* IQR for both sides. The diamond in each subplot is the outlier.

When stratifying the 361 patients based on the diagnosis on admission, 24 patients had congestive heart failure (CHF), 29 patients had cerebrovascular accident (CVA), 29 patients had chronic obstructive pulmonary disease (COPD), 53 patients had chronic kidney disease (CKD), and 67 patients had arrhythmias. Figure 7 shows the segmentation of “High” or “Urgent” alerts in each physiological parameter among these groups of patients when using the MPRT-WS tool.

Lastly, we analyzed the sensitivity and specificity at a 15-min monitoring interval of the MPRT-WS tool and found that there were 88,977 (93.7%) “Low” events, 5,467 (5.8%) “Medium” events, and 521 (0.5%) were “High” risk events. 28 out of 39 patients that deteriorated received an “Urgent” score, and 22 of them were identified 43.3 ± 50 h before deterioration. The sensitivity was 56.4%.

## Discussion

We present a new pre-symptomatic and real-time detection and warning tool, based on nine physiological parameters that are frequently monitored using a wearable monitoring platform. We show that the platform allows for early detection of high-risk deterioration more than 40 h before such deterioration occurs.

Currently-used EWS systems have relatively high sensitivity yet low specificity, resulting in multiple alerts and often leading to alarm fatigue. Moreover, EWS alerts do not necessarily lead to actual deterioration, as was previously demonstrated (Itelman et al., 2022), and it was not designed to provide a real-time alert of actual deterioration. The EWS scores are calculated infrequently, either once a day or once a shift. The NEWS score was formulated in 2012, aimed to standardize the method of recognizing deteriorating patients and intensifying care through widespread teaching and training (Smith et al., 2013; Nagarajah et al., 2022). It was shown to have a greater value in discriminating patients at risk than 33 other EWS systems (Smith et al., 2013), and soon after was adopted around the world. It is the common EWS tool used today in Israel, and for this reason we used it in the current study. A few years later, an updated version of the score with modified considerations regarding the vitals, added confusion and oxygen saturation scale for patients with type 2 respiratory failure, was defined as the NEWS2. NEWS2 has been recently widely used during the COVID-19 pandemic, and shown to be superior to scores directed at infections and sepsis (Myrstad et al., 2020). Both methods have been extensively validated in the literature (Alam et al., 2015; Bilben et al., 2016; Ehara et al., 2019; Nagarajah et al., 2022), and adapted for various settings and populations (Monaghan, 2005; Lee et al., 2020). Another adaptation of the input criteria of NEWS resulted in the Modified Early Warning Score (MEWS), in which other parameters are also added (Cooksley et al., 2012; Young et al., 2014). In all of these studies, the scores were calculated infrequently by the healthcare providers. In recent years, efforts were put in building scores that incorporate temporal characteristics and advanced analytics (Shamout et al., 2020; Zhu et al., 2020). Though showing promise, none have been fully validated and implemented yet.

In the study by Itelman et al. (2022), the authors concluded that frequent measurement of NEWS values had no added value in terms of earlier detection of risk or improvement of the sensitivity and specificity, over the infrequent measurement of the NEWS score. An anticipated step would be to use the parameters of NEWS only and add the temporal characteristics we developed. This would allow to assess the added value of the additional vital parameters, which have been included to MPRT-WS. In our hands, such an assessment resulted in lower sensitivity of the score in identifying deteriorating patients, emphasizing the importance of including the additional parameters. Moreover, we aimed to develop a new score based on real-time frequent measurements, providing an advanced multi-parameter-based and real-time detection of patients’ deterioration, on top of the warning score tool.

The novel concept implemented in this work is the immediate detection and warning component, based on the definition of an “urgent” score. The system detected 25,635 readings (21.8% of the total readings) as “Urgent”. Unlike the NEWS score, this provides real-time telemetry-like data, about an ongoing clinical deterioration that should be cared for at that moment by the healthcare providers. As such, it is not an EWS, but rather a real-time alert tool. Though the percentage is high, the patients included in the study were all complex and multi-morbid, defined on admission by the attending physician as those that have a high risk to deteriorate within the first 72 h after admission. Thus, this result is not surprising, especially as within this study, healthcare providers did not respond to the alert system, allowing for repeated or continuous “Urgent” alarms to keep appearing, a situation that we assume would not occur in a real-world setting. As the MPRT-WS tool differentiates between an urgent score and lower levels of scores that do not represent an imminent danger, it could help target events that necessitate an immediate response, versus clinical conditions that while may still lead to deterioration, should not be regarded as immediate in nature. As a result, in a real-life setting, this could help in reducing alarm fatigue that might develop when using an EWS system integrated with frequent telemetry monitoring.

The addition of physiological parameters can improve the clinical understanding of the medical condition of any given patient. Thus, the MPRT-WS tool might provide a better scale, with fewer false alerts. When adding the time dimension to the level of risk, healthcare providers can now get a timely alert, helping them as a clinical decision-support tool when providing treatment to patients at high risk. Moreover, this tool allows the healthcare providers to better manage their time, as they receive timely alerts while performing all other duties on the ward. By using a system that provides frequent or intermittent monitoring, the concept of EWS systems could shift into better-managed care, focusing on pre-symptomatic detection. This still needs to be validated in a real-world setting in which the monitoring data provided by the platform is readily available to all healthcare providers.

When analyzing a 15-min monitoring interval of the MPRT-WS we found reduced sensitivity, with less patients identified early. Theoretically, when adding to that a potentially life-threatening deviation in vitals, a 15-min interval within the risk score might prevent the system from providing a timely alert. For these reasons, we think that the 10-min interval is the adequate interval for the novel MPRT-WS tool. This aspect should be further studied.

Though there are multiple EWS systems in clinical practice, most healthcare providers need to calculate the scores, commonly resulting in incorrect values (Wuytak et al., 2017). This is another advantage of the current platform, as it automatically calculates the score. Furthermore, in this study the NEWS score was calculated at a higher frequency than in other settings. The combination of these capabilities has the potential to assure better response in real-world scenarios.

A limitation of this study is the need to perform a large-scale validation study, preferably comparing it with other currently-used tools, assuring our assumptions regarding the pre-defined parameter cutoffs and capabilities of the new tool indeed provides a better pre-symptomatic and real-time detection and warning tool. Moreover, for now, it has no consideration of oxygen supplementation or the level of consciousness of the individual (=AVPU). These might be less relevant in frequently calculated scores, yet this still needs to be proved in a relevant clinical setting.

By looking at the segmentation of alerts in the various physiological parameters after stratifying the patients into the different clinical groups (Figure 7), we were trying to see whether it would be possible to aim at a tailored warning score for different medical conditions. As can be seen, and even though it is preliminary, for each medical condition the “High” and “Urgent” values of the various parameters provides a different pattern. This is encouraging, as it offers support for future efforts in studies with larger cohorts, to develop disease- and patient-tailored scores, with emphasis on pre-symptomatic detection and prevention in numerous patient sub-populations.

Another future aim would be to show the potential of such an advanced real-time alert system to reduce alarm fatigue by increasing the specificity using multiple cardio-respiratory vitals.

## Conclusion

As the developed MPRT-WS tool is based on continuous monitoring capabilities, it has the potential to allow healthcare providers to better manage their time, as they receive timely alerts of deterioration and the risk for deterioration of patients under their direct care. This could lead to a paradigm shift when compared with current EWS systems.

## Data Availability

The data supporting the conclusion of this article will be made available by the authors, without undue reservation.
