# Supplementary material for: Developing a real-time detection tool and an early warning score using a continuous wearable multi-parameter monitor
Source: Front Physiol. 2023 Mar 29;14:1138647. doi: 10.3389/fphys.2023.1138647 (PMC10090377; doi:10.3389/fphys.2023.1138647)

Supplemental Figure 1. An example of analyzing and building the score of an individual parameter. Stroke volume (SV) measurements were analyzed in two populations: green bars – healthy individuals; red bars – hospitalized patients. The normal range of SV values was adopted from what is accepted in the literature and was defined as "0" (60-100 mL/beat). Next, by combining data collected among both groups, the expert panel defined the range for each of the other levels. Level "1" – 51-60 mL/beat and 101-120 mL/beat. Level "3" – 50 mL/beat and below and 121 mL/beat and above. For SV, Level "2" was not defined as the range is too small and the clinical implications especially in the lower range are more significant.

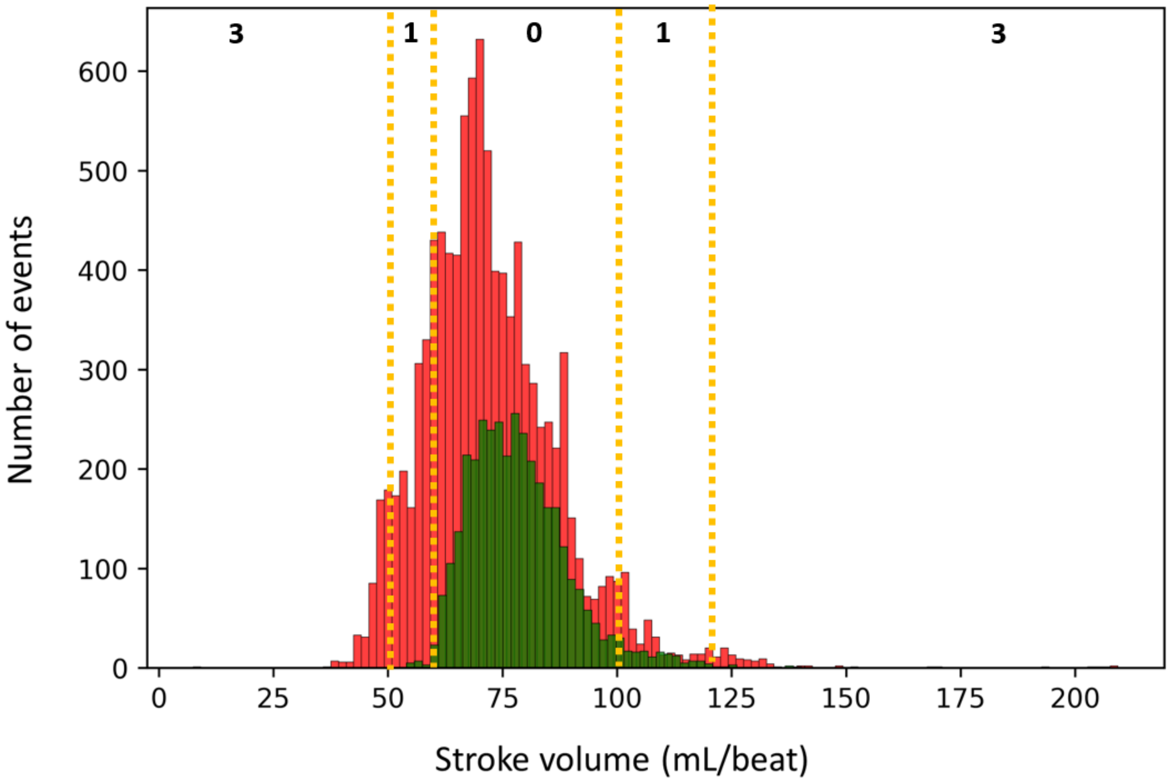

Supplement: Supplementary file 1 [file DataSheet1.pdf]
